# Supplementary material for: Digital Health Interventions to Improve Adolescent HPV Vaccination: A Systematic Review
Source: Vaccines (Basel). 2023 Jan 22;11(2):249. doi: 10.3390/vaccines11020249 (PMC9963303; doi:10.3390/vaccines11020249)
Supplement: Supplementary file 1 [file vaccines-11-00249-s001.zip › vaccines-2115461-supplementary.pdf]

Supplementary Materials

Table S1: Cochrane risk of bias assessment tool for RCTs

Author	Risk of bias (high, low, unclear)						
	Selection bias		Performance bias	Detection bias	Attrition bias	Reporting bias	Other bias
	Random sequence generation	Allocation concealment	Blinding of participants and personnel	Blinding of outcome assessment	Incomplete outcome data	Selective reporting	
Pot et al., 2017	Unclear	Unclear	High	Unclear	High	Low	
Rand et al., 2017	Unclear	High	High	low	Low	Low	
Ortiz et al., 2018	Unclear	Unclear	Unclear	High	Unclear	Low	
Dempsey et al., 2019	Low	Low	Low	High	High	Unclear	Social desirability bias
Dixon et al., 2019	Low	unclear	High	High	Low	Low	
Richman et al. 2019	Unclear	Unclear	Low	High	High	Low	
Tull et al., 2019	Low	Low	Low	High	Low	Low	
Cates et al., 2020	Low	Unclear	Unclear	High	High	High	
Panozzo et al, 2020	Unclear	Unclear	Unclear	High	High	Low	
Buller et al., 2021	Low	Low	Low	High	High	Unclear	Generalizability bias
Chodick et al. 2021	Unclear	Unclear	Unclear	High	Unclear	Low	
Woodall et al., 2021	Unclear	Low	Low	High	Low	Unclear	
Wynn et al., 2021	Low	Unclear	High	Low	Low	Unclear	
Marshall et al., 2022	Unclear	unclear	Unclear	High	Unclear	unclear	
Shegog et al., 2022	Unclear	High	High	High	High	Low	

Table S2: Risk of bias assessment for non-randomized studies: The National Institute of Health (NIH) quality assessment tool for pre-post study with no control group

Major components (Yes, No, Not reported/Not able to determine/Not applicable)	Author			
	Chen et al., 2019	Sundstrom et al., 2021	Occa et al., 2022	Chen et al., 2022
1. Was the study question or objective clearly stated?	Yes	Yes	Yes	Yes
2. Were eligibility/selection criteria for the study population prespecified and clearly described?	Yes	Yes	Not reported	Yes

3. Were the participants in the study representative of those who would be eligible for the test/service/intervention in the general or clinical population of interest?	Yes	No	Yes	No
4. Were all eligible participants that met the prespecified entry criteria enrolled?	No	No	Yes	Yes
5. Was the sample size sufficiently large to provide confidence in the findings?	No	No	No	Yes
6. Was the test/service/intervention clearly described and delivered consistently across the study population?	Yes	Yes	Yes	Yes
7. Were the outcome measures prespecified, clearly defined, valid, reliable, and assessed consistently across all study participants?	Yes	Yes	Yes	Yes
8. Were the people assessing the outcomes blinded to the participants' exposures/interventions?	Not reported	Not reported	Not reported	Not reported
9. Was the loss to follow-up after baseline 20% or less? Were those lost to follow-up accounted for in the analysis?	Yes	Not reported	Not reported	Not applicable
10. Did the statistical methods examine changes in outcome measures from before to after the intervention? Were statistical tests done that provided p values for the pre-to-post changes?	Yes	Yes	Yes	Yes
11. Were outcome measures of interest taken multiple times before the intervention and multiple times after the intervention (i.e., did they use an interrupted time-series design)?	No	No	No	No
12. If the intervention was conducted at a group level (e.g., a whole hospital, a community, etc.) did the statistical analysis take into account the use of individual-level data to determine effects at the group level?	Not applicable	Not applicable	Not applicable	Not applicable
Quality Rating (good/fair/poor)	Good	Fair	Fair	Good

Table S3: Summary of Findings

Study	Setting	Technology	Target participants	Aim	Study design/intervention	Theory-based?	Outcome measures	Results
Pot et al., 2017	Netherlands	Website	N=8062 mothers n=3995 (intervention) n=4067 (control)	To evaluate the effectiveness of the web-based tailored intervention for promoting HPV vaccination acceptance by means of a RCT	RCT; the website provided tailored feedback from two virtual assistants and consisted of four menu options: 1) two-sided information (risk of contracting an HPV infection and the risks and effectiveness of the vaccine) about HPV vaccination; 2) a decisional balance weighing perceived pros and cons of vaccination; 3) practical background information including clinic locator and how to talk to child about HPV vaccination; 4) frequently asked questions	IM protocol	HPV vaccine uptake; vaccination intention; attitudes and beliefs about HPV vaccination	Mothers in the intervention group had more knowledge, were more informed, were more intended to vaccinate their daughter and more positive attitudes and beliefs about HPV vaccination. Effect sizes were small. No effect was found on uptake.
Rand et al., 2017	USA	Text messages	N=749 parents of 11- to 17-year old n=178 phone intervention (180 control) n=191 text intervention (200 control)	To assess the effect of phone or text message reminders to parents of adolescents on HPV vaccine series completion	Parallel RCT; a maximum of three successful reminders for each dose (1 week apart) was sent to parents in the phone and text intervention groups. If messages were not successful, up to six attempts were made to reach the participant.	NA	Time to receipt of the third dose of HPV vaccine; HPV vaccination rates	Text reminders for HPV vaccination significantly decreased the adolescents' time to receipt of subsequent HPV vaccination, whereas phone reminders were effective for those enrolled at dose 1 but not at dose 2; 18% more adolescents in the text intervention compared with control had received all three vaccine doses
Mohanty et al., 2018	USA	Social media: **3forME**	N= 2106 adolescents aged 13-18 years	To assess the campaign reach, engagement, and HPV vaccine uptake among adolescents through the Facebook campaign	The Facebook campaign had six advertising themes to prompt adolescents to seek HPV vaccination: 1) *Ownership Over One's Own Health* focused on self-determination and being independent; 2) *Back to School* focused on timeliness and routine healthcare; 3) *Disease Risk* focused on HPV disease, risk reduction, and prevention strategies; 4) *Peer Support* focused on preventing transmission and protecting others; 5) *Summer Protection*	HBM	Reach, engage; HPV vaccine uptake	Only 2 adolescents were vaccinated as a result of the Facebook campaign. The campaign was well-received, far-reaching and generated awareness and conversations among adolescents but did not appear to be a sufficient driver for HPV uptake.

					focused on staying safe during the summer months; 6) *Philadelphia* focused on sense of community and local services.			
Amresh et al., 2019	USA	Game: **We Care-Teen**	N=8 parent-adolescent (11-14 years) dyads	To develop and pilot test a game-based intervention for adolescents aged 11-14 and their parents to increase uptake of HPV vaccine in clinical settings	Sequential mixed-methods research design (pilot test); the game includes tailored feedback addressing knowledge, barriers, and facilitators. Design features include identity (become game character via avatar), control (ability to influence story), feedback (receive information about efficacy of actions), interactivity (initiate actions and receive evaluative information about actions), and immersion (sense of presence, transportation or integration within the game)	SCT	Qualitative feedback about the design and development of the game-based intervention to increase vaccine uptake	The design of the game should accommodate the vast difference in how boys, girls and parents approach games; flexible and customized based on player characteristics. Game should be short and to the point to accommodate short attention span
Dixon et al., 2019	USA	Digital video	N=1596 parents of adolescents aged 11-17 years n=537 (intervention) n=1059 (control)	To test the effect of a digital HPV vaccine educational intervention	RCT; parents watched a digital video outlining the risks and benefits of vaccine using a tablet in the examination room.	TPB	HPV vaccine uptake (change in vaccination status)	The proportion of adolescents with an observed change in vaccine status was higher for those attending an intervention clinic (64.8%) vs. control clinic (50.1%). Adolescents whose parents watched the video had a 3-times greater odds of receiving a dose of the HPV vaccine.
Richman et al., 2019	USA	Text messages	N=257 parent-child dyads n=129 dyads (intervention) n=128 dyads (control)	To determine if electronic reminders and educational messages can increase HPV knowledge among parents and increase HPV vaccine uptake among low-income adolescents	RCT; parents in the intervention group received four health education messages about HPV and the vaccine, two appointment reminder messages and one message asking participants to take the follow-up survey once per month across seven months. Parents in the control group received a paper card that tells them when to return for the second and third doses.	NA	HPV-2 and HPV-3 uptake; HPV and HPV vaccine knowledge	Completion rates for intervention and control groups were similar for HPV dose 2 (65% vs.65%) and HPV dose 3 (35%v vs. 30%), respectively. Knowledge change was higher for the intervention group but this difference was not statistically significant. Electronic reminders to promote vaccine completion were not effective and did not increase HPV and HPV vaccine knowledge at statistically significant level.
Ruiz-Lopez et al., 2019	Norway	Mobile Game: **FightHPV**	N=23 adolescents (10 girls, 13	To describe the development of and evaluate a	Focus group; FightHPV is a game-based learning tool communicates concepts about HPV, associated diseases and their prevention	SCT, social nudging	HPV-related knowledge; general	A significant increase in HPV knowledge was observed after playing the game.

			boys) aged 16-18 years old	mobile app	by representing relationships among 14 characters in 6 episodes of 10 levels each, with each level being represented by a puzzle. Main concepts were reinforced with short messages of text explanations.		(qualitative) feedback on the mobile game	
Tull et al., 2019	Australia	Text messages	N=4386 parents of consented adolescents n=1442 (motivational message group) n=1418 (self-regulatory message group) n=1526 (control group)	To test the hypothesis that sending a SMS reminder to parents would lead to greater uptake of the vaccine within the program	Multi-arm parallel RCT; two groups received SMS messages with either motivational (highlighting susceptibility) content or self-regulatory (implementation intentions) content and one group did not receive any text message reminders.	Motivational strategy based on HBM	vaccine uptake irrespective of the dose of the vaccine; vaccine completion	85.71% students in the control condition received the vaccine, compared with 88.35% in the motivational message condition, and 89.00% in the self-regulatory message condition. Both intervention messages were similarly effective.
Cates et al., 2020	USA	Game: **Land of Secret Gardens**	N=47 parent-preteen (11-12 years) dyads n=21 (intervention) n=26 (control)	To evaluate the acceptability and feasibility of using a serious video game about HPV vaccination to promote conversations about and decisions to seek HPV vaccination	RCT; preteens played the game and completed 3 tasks in the video game. Preteens were exposed to messages about HPV and the HPV vaccination throughout the game, and continued use of the game would result in greater message exposure. Instructions for parents to guide game play were posted on the parental portal, along with a video that described the background and premise of the game.	SDT	HPV-related knowledge, vaccination self-efficacy; vaccination rate; qualitative feedback	The vaccination self-efficacy score was higher in the comparison group than the intervention group (1.65 vs. 1.45). The overall mean decisional balance score trended toward greater support of vaccination, although differences between the groups were not significant. Vaccine initiation and completion rates were higher in the intervention group (22% vs. 15%) than in the comparison group (9% vs. 2%), although the difference was not significant.
Chen et al., 2020	USA	Computer-tailored educational intervention	N= 29 parents of children aged 11-17	To investigate the preliminary efficacy of the intervention on	One-group pre, post-test quasi-experimental design; the intervention was delivered to parents via tablet computers and it featured an avatar who provided tailored messages to	HBM, TPB	HPV-related knowledge; vaccination intention;	Parents had significant positive changes in HPV-related knowledge and facilitators over time. Nearly all (97%) of the

			years	parental intentions to vaccinate their child, and action to obtain the first HPV vaccine	participants addressing knowledge, perceived risks, facilitators, barriers, and cultural norms regarding HPV vaccination.		vaccine uptake (whether or not the target child received the first dose of HPV vaccines).	parents reported that the intervention helped them make a decision about their child's HPV vaccination. 100% of parents intended to get their children vaccinated but the actual vaccination behavior was unknown.
Teitelman et al., 2020	USA	app: **Vaccipack**	N=54 parents and adolescents n=34 parents n=20 adolescents aged 11-14 years	To describe the development, acceptability, and intention to use the mobile app	Single group pre-posttest design; Vaccipack, was designed to promote uptake and completion of the adolescent HPV vaccine series. The app included an introductory video (basic information about HPV), reminders about second dose, inspirational personal stories about parents (common parental beliefs), frequently asked questions, and discussion forum with tags to identify the topic	IBM, TAM	App acceptability; intention to use the app; behavioral beliefs and self-efficacy beliefs about using the app	75% of adolescents and 88% of parents intended to use the app in the next 2 weeks. Acceptability of the app was high among both groups: 88% of parents and 75% of adolescents indicated that the app was easy to use, and 82% of parents and 85% of adolescents perceived the app to be beneficial. Higher levels of app acceptability were found among parents with strong intentions to use the app.
Chodick et al., 2021	Israel	social media	N= 21592 mothers of 14-year-old daughters n=17271 (intervention) n=4321 (control)	To assess whether a targeted Facebook campaign among mothers may increase the uptake of HPV immunization among their 8th-grade daughters	Randomized field study; the Facebook campaign was designed to introduce content and generate awareness of HPV vaccination program. The campaign material had been prepared by gynecologists and were deployed to study participants through Facebook news feed. Specific barriers to action were addressed in short videos and textual posts. Messages provided audiences with a forewarning of counterarguments followed by refutations of these counterarguments. Other campaign messages addressed additional issues and concerns regarding HPV vaccine hesitancy, HPV prevalence and safety issues.	Inoculation theory	Effectiveness of the campaign; HPV immunization history among daughters of the study participants	The uptake of HPV vaccine among daughters of women allocated to the intervention arm (55.3%) was similar (p=0.749) to 55.0% in the control group. The intervention significantly reduced vaccine uptake (35.0% vs. 39.0%) in the lowest SES quartile, while it increased vaccine uptake from 52.6% to 55.8% in the second SES quartile. Among mothers in higher SES levels, daughters of exposed and unexposed mothers had similar immunization rates.
Sundstrom et al., 2021	USA	Social media	N=22 parents	To develop and test a collaborative online learning environment to	One-group pre-test posttest design; content was delivered through a private Facebook group, bi-weekly emails with facts about HPV vaccination, and two online webinars about HPV vaccination, addressing common	HBM, TTM	Knowledge of HPV and the HPV vaccine; attitudes and beliefs about	Following the intervention, two participants changed from being unsure to planning to vaccinate at the recommended age but there were no statistically significant

				increase HPV vaccination by training and supporting parents to serve as proponents and social media champions to overcome barriers to HPV vaccination.	misconceptions and how to be an effective spokesperson for HPV vaccination. Participants responded to polls, posted messages, and asked questions of one another, as well as experts on the research team.		HPV and the HPV vaccine; behavior/behavioral intention regarding HPV and the HPV vaccine.	differences between pretest, posttest and 6-month follow-up responses within participants. The difference in knowledge of HPV and HPV vaccination between pretest and posttest was significant. The intervention increased parents' confidence and motivated them to speak more freely about HPV vaccination in-person and online with others in their social networks.
Woodall et al., 2021	USA	app: **Vacteens.org**	N=82 parent-adolescent (11-14 daughters) dyads in nine pediatric clinics	To test a mobile web app for parents and their adolescent daughters encouraging HPV vaccination	Clinic-cluster randomized trial; the app had five modules: 1) *Get Answers!*: information about HPV and vaccines, addressing misinformation about the vaccine; 2) *Let's Talk*: a video simulation of how to talk with your child about HPV vaccination; 3) *Vaccine How-To*: instructions for making an HPV vaccination appointment; 4) *Teen Tools*: interactive and engaging activities for teens and parents to motivate vaccination; 5) *We're Ready*: reminder systems to promote completion of the vaccination series.	IDM, DIT	HPV and HPV vaccination knowledge; beliefs about HPV and HPV vaccination; intention to vaccinate	At 3-month, parents using the app had higher positive HPV vaccine beliefs, intent to vaccinate and vaccine confidence than those who did not use the app. The Vacteens.org group had higher first dose HPV vaccination (59.4% vs. 40.6%) and higher vaccination series completion (68.4% vs. 31.6%) than the usual care group
Wynn et al., 2021	USA	Text messages	N=956 parents of adolescents aged 9-17 years n=475 (intervention text message arm) n=481 (conventional text message arm)	To compare the impact of precision SMS text message on HPV vaccine series completion with conventional SMS text message reminders in a RCT	Parallel RCT; precision text messages included stage-targeted educational information, next dose due date, and site-specific walk-in hours. Conventional text messages did not include educational information.	TTM	HPV vaccine series completion rates within 12 months	Adolescents in either SMS text message arm had significantly higher completion rates than controls but educational information did not provide an added benefit to this population as the precision and conventional text message arms had similarly high series completion rates (72.4% vs. 75.7%).

Chen et al., 2022	USA	Digital storytelling	N=114 Vietnamese American or Vietnamese immigrant mothers of unvaccinated children aged 11-14 years	To examine the effects of the a digital storytelling intervention on intention to vaccinate children among VA mothers of unvaccinated children	Single-group quasi-experimental pretest-posttest design; the digital storytelling intervention consisted of two stories (3 minutes each) codeveloped by VA mothers of vaccinated boys and girls. In the stories, the mothers described initial worries about the misinformation in social media and seeking information from reliable resources. Each participant was invited to view the digital stories and immediately complete online postintervention questionnaire.	NA	Intention to vaccinate children against HPV	Mothers' intention to vaccinate their children increased from 53% to 74% after the intervention; the difference was large and statistically significant. Scores on qualitative assessment scale were high, suggesting high levels of identification and engagement with the stories.
Kim et al., 2022	USA	app: **Vax4HPV**	N=15 parents with at least one child aged 9-14 years old	To explore the desired content, designs, and features of a Vax4HPV smartphone app using a theory-based, user-centered approach	Focus group; the app provides an urgent and strong, tailored HPV vaccine recommendation, HPV information, an activity, a clinic locator and a chat. The app prototype provides parents with an exemplar script to help parents communicate with their child.	Information systems research framework	Qualitative feedback; app usability	Parents suggested that the app provide tailored yet gender-neutral information designed to eliminate parental vaccine hesitancy, include a simplified and interactive dashboard user interface and use nudging through social media networks to attract more end users
Marshall et al., 2022	Ireland	Online video: **"Is the HPV vaccine for me?"**	N=35 parent-daughter dyads<br>n=18 (intervention)<br>n=17 (control)	To design, develop and evaluate the feasibility of a theory- and evidence-based intervention to improve HPV and HPV vaccine knowledge and intention to vaccinate among parent-daughter dyads	RCT; In the 6-minute video, a narrative approach was applied, mapping the adolescent HPV vaccine decision journey. Definitions and numerical information were complemented by graphical illustration. The video finished with a reminder that most girls in Ireland accept the HPV vaccine.	TDF	HPV and vaccine knowledge; intention to vaccinate	The intervention resulted in a statistically significant increase in HPV and HPV vaccine knowledge and intention to vaccinate. All intervention participants found the video interesting, while 96% found it useful.
Occa et al., 2022	Italy	Video and game: **Salute e HPV**	N=35 children (11-12 years)<br>n=20 (video group)	To assess the feasibility of using an evidence-based animated video	One-group pre-post; the animated video (Salute e HPV) highlights that children need to take the HPV vaccine to remain healthy; health care professional describes HPV and associated diseases; focuses on the safety of	TPB, SCT	Knowledge of HPV; perceptions (attitudes and beliefs) about	Both the animated video and a web-based game increased children's knowledge and positive perceptions about HPV and HPV vaccination. Any single message

			n=15 (game group)	and a web-based game to help children participate in discussions about HPV vaccination and improve several HPV-related outcomes	the vaccine; describes the procedure for getting the vaccine; explains that the vaccine is available for free and recommends HPV vaccination conversation. The game is a web-based quiz with content consistent with the content of the video. The game was designed to include several motivational affordances (points and achievement, progress bar, clear goal, feedback)		HPV and HPV vaccination; qualitative feedback	was not more effective than the others. Qualitative feedback: the children discussed aspects of the features and characters they liked and those that need improvements.
Shegog et al., 2022	USA	app: **HPV CancerFree (HPVCF)**	N=375 parent-children (10-17 years) dyads n= 168 (intervention) n=207 (control)	To determine if parents of vaccine-eligible youth who use the app HPVCF demonstrate greater HPV vaccination rates improved intention to vaccinate, improved attitudes	RCT; the HPVCF app had four components: 1) *HPV A-Z* providing facts about HPV and HPV vaccine; 2) *Bust a Myth*, addressing HPV vaccination barriers; 3) *Notes 4 Doc,* a medium to facilitate communication with providers on HPV vaccine; 4) *Get the Vax*, enabling parents to schedule tailored HPV vaccination appointment reminders	HBM, SCT, TRA	HPV and HPV vaccination knowledge, attitudes and beliefs about HPV vaccination, Intention to vaccinate, vaccine initiation	Parents assigned to receive HPVCF significantly increased knowledge about HPV and HPV vaccination and perceived effectiveness of the vaccine. Change in HPV vaccine initiation was not significant.

TDF: Theoretical Domains Framework
IDM: Informed Decision Making
DIT: Diffusions of Innovation Theory
TAM: Technology Acceptance Model
IBM: Integrated Behavioral Model
SDT: Self-determination Theory
IM: Intervention Mapping
HBM: Health Belief Model
TPB: Theory of Planned Behavior
SCT: Social Cognitive Theory
TTM: Transtheoretical Model

TRA: Theory of Reasoned Action
